# Identifying Women at High Risk of 90 Day Death after Elective Open Abdominal Aortic Aneurysm Repair: A Multicentre Case Control Study

**DOI:** 10.1016/j.ejvsvf.2022.10.005

**Published:** 2022-11-09

**Authors:** Victoria N. Tedjawirja, Ruth M.A. Bulder, Jan H.N. Lindeman, Jaap F. Hamming, Susan van Dieren, Ron Balm, Mark J.W. Koelemay, Study Group Collaborators, G.P. Akkersdijk, G.J. Boer, L.H. Bouwman, J. Diks, J.W. Elshof, R.H. Geelkerken, G.H. Ho, P.T. den Hoed, B.P. Keller, J.W. Klunder, O.H. Koning, R.R. Kruse, J.H. Lardenoye, M.S. Lemson, S.J.G. Leeuwerke, F.T. van der Linden, M.E. Pierie, H.P. van ’t Sant, O. Schouten, S.M. Schreuder, R.M. The, L. van Silfhout, R.B. van Tongeren, P.W. Vriens, A.M. Wiersema, A. Wiersma, C.J. Zeebregts

**Affiliations:** c Department of Surgery, Maasstad Hospital, Rotterdam, the Netherlands; d Department of Surgery, Zuyderland, Sittard-Geleen, Heerlen Brunssum, the Netherlands; e Department of Surgery, Bravis Hospital, Bergen op Zoom Roosendaal, the Netherlands; f Department of Surgery, VieCuri Hospital, Venlo Venray, the Netherlands; g Department of Vascular Surgery, Medisch Spectrum Twente, Enschede, the Netherlands; h Multi-Modality Medical Imaging Group, Techmed Center, University of Twente, Enschede, the Netherlands; i Department of Surgery, Amphia Hospital, Breda, the Netherlands; j Department of Surgery, Ikazia Hospital, Rotterdam, the Netherlands; k Department of Surgery, Martini Hospital, Groningen, the Netherlands; l Department of Surgery, St. Jansdal Hospital, Harderwijk, the Netherlands; m Department of Surgery, Jeroen Bosch Ziekenhuis, ’s Hertogenbosch, the Netherlands; n Department of Surgery, Ziekenhuis Groep Twente, Hengelo Almelo, the Netherlands; o Department of Surgery, Rijnstate Hospital, Arnhem, the Netherlands; p Department of Surgery, Slingeland Hospital, Doetinchem, the Netherlands; q Department of Surgery, St. Anna Hospital, Geldrop Eindhoven, the Netherlands; r Department of Surgery, Isala Hospital, Zwolle, the Netherlands; s Department of Surgery, OLVG, Amsterdam, the Netherlands; t Department of Surgery, Reinier de Graaf Gasthuis, Delft, the Netherlands; u Department of Radiology, Amsterdam University Medical Center, University of Amsterdam, Amsterdam, the Netherlands; v Department of Surgery, Deventer Hospital, Deventer, the Netherlands; w Department of Surgery, Elizabeth TweeSteden Hospital, Tilburg, the Netherlands; x Department of Surgery, Dijklander Hospital, Purmerend, the Netherlands; y Department of Surgery (Division of Vascular surgery), University Medical Center of Groningen, University of Groningen, Groningen, the Netherlands; a Amsterdam UMC location University of Amsterdam, Department of Surgery, Amsterdam Cardiovascular Sciences, Amsterdam, the Netherlands; b Leiden University Medical Centre, Department of Vascular Surgery, Leiden, the Netherlands

**Keywords:** Abdominal aortic aneurysm, Mortality, Risk factors, Surgery, Women

## Abstract

**Objective:**

The aim of this study was to identify risk factors for 90 day death after elective open surgical repair (OSR) of abdominal aortic aneurysms (AAAs) in women.

**Methods:**

This was a multicentre case control study. The nationwide Dutch Surgical Aneurysm Audit registry (2013–2019) was solely used to identify women who underwent elective OSR as eligible patients. Data for this study were subsequently collected from the patients’ medical files. Women with AAA were included and those who died (cases) were compared with those who survived (controls) 90 days after surgery. Inflammatory, mycotic, or symptomatic or ruptured AAA were excluded. The association between pre- and peri-operative risk factors and death was assessed by logistic regression analysis in the whole sample and after matching cases to controls of the same age at the time of repair. Mesenteric artery patency was also assessed on pre-operative computed tomography and used in the analysis.

**Results:**

In total, 266 patients (30 cases and 236 controls) from 21 hospitals were included. Cases were older (median [interquartile range; IQR] 75 years [71, 78.3] *vs.* 71 years [66, 77]; *p* = .002) and more often had symptomatic peripheral arterial disease (PAD) (14/29 [48%] *vs.* 49/227 [22%]; *p* = .002). Intra-operative blood loss (median [IQR] 1.6 L [1.1, 3.0] *vs.* 1.2 L [0.7, 1.8]), acute myocardial infarction (AMI) (10/30 [33%] *vs.* 8/236 [3%]), renal failure (17/30 [57%] *vs.* 33/236 [14%]), and bowel ischaemia (BI) (17/29 [59%] *vs.* 12/236 [5%]) were more prevalent among cases. Older age (odds ratio [OR] 1.11, 95% confidence interval [CI] 1.03–1.19) and PAD (OR 3.91, 95% CI 1.57–9.74) were associated with death. Multivariable analysis demonstrated that, after adjustment for age, AMI (OR 9.34, 95% CI 1.66–52.4) and BI (OR 35.6, 95% CI 3.41–370) were associated with death. Superior mesenteric artery stenosis of >70% had a clinically relevant association with BI (OR 5.23, 95% CI 1.43–19.13; *p* = .012).

**Conclusion:**

Age, symptomatic PAD, AMI, and BI were risk factors for death after elective OSR in women. The association between a >70% SMA stenosis and BI may call for action in selected cases.

## Highlights

• Identifying risk factors for death after elective open abdominal aortic aneurysm repair in women.
• Older age and symptomatic peripheral artery disease are associated with death.
• Acute myocardial infarction and bowel ischaemia (BI) also increase mortality risk.
• Superior mesenteric artery stenosis seems to be a clinically relevant factor for BI.

## Introduction

A recent meta-analysis of 13 observational studies found an increased risk of death after open surgical repair (OSR) of abdominal aortic aneurysm (AAA) in women *vs.* men (odds ratio [OR] 1.49, 95% confidence interval [CI] 1.37–1.61).^1^ Even after multivariable adjustment for risk factors, female sex is associated with higher peri-operative mortality.^2^, ^3^, ^4^ It has been suggested that differences in the recognition of pre-operative risk or complications may account for the increased mortality, yet it is largely unexplained.^1^ In a systematic review comparing baseline comorbidity between women and men undergoing elective OSR for AAA, it was found that women were significantly older at the time of surgery and had diabetes and ischaemic heart disease less often.^5^ As the prevalence of AAA is much higher in men than women, the current knowledge of risk factors may be primarily applicable to men.^6^

The peri-operative mortality rate is 7.3% in women who undergo elective OSR of AAA in the Netherlands.^2^ The high mortality rate in women calls for studies to elucidate factors that are particularly relevant in women. In a previous study, the causes of death in women after elective OSR were investigated and it was found that the majority died from bowel ischaemia (BI) and myocardial infarction (MI).^7^ However, the study design prevented identification of risk factors for death, including those not attributed to BI or MI. Therefore, a case control study was carried out with the aim of identifying risk factors for the all cause 90 day mortality rate after elective OSR of AAA in women. The aim was to identify women at high risk, so that pre-operative management might be optimised to improve post-operative survival, or the indication for elective OSR reconsidered.

## Materials and methods

### Ethical regulations

The Medical Ethics Committee of the Amsterdam University Medical Centre (Amsterdam UMC, University of Amsterdam) determined that this study was not subject to the Dutch Medical Research Involving Human Subjects Act (W20_389 # 20.433). The study complied with the local regulations of the participating medical centres and was approved by the local ethics committees. A data sharing agreement was obtained between the participating centres and the Amsterdam UMC.

Dependent on local regulations, patients were either informed about the study and asked for written informed consent or sent a letter with the option to reply within four weeks if they objected to their medical records being used for this study. Patients who did not give informed consent or objected to the use of their records were excluded. In accordance with the Dutch General Data Protection Regulation, an informed consent procedure was not applicable to deceased patients. Patient data were pseudonymised and stored in Castor EDC (an online database and data management tool), protected against unauthorised access in accordance with the Good Clinical Practice Guidelines.

### Study design and population

**Figure 1:**
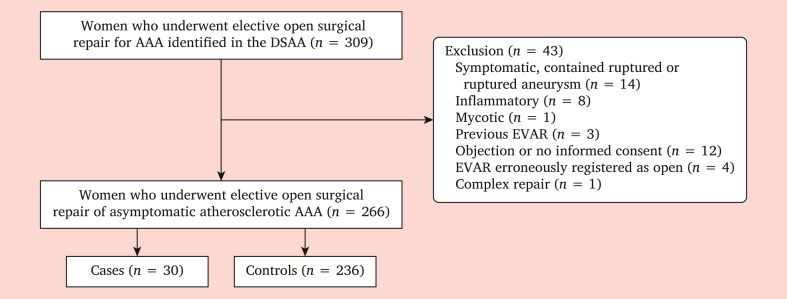
Inclusion of the study population of women who underwent primary elective open surgical repair of asymptomatic abdominal aortic aneurysm (AAA) in the Netherlands from 21 centres (2013-2019). DSAA = Dutch Surgical Aneurysm Audit.

All women who underwent elective OSR of an AAA in the Netherlands between January 2013 and December 2019 were eligible for this study. The Dutch Surgical Aneurysm Audit (DSAA) registry was solely used to identify these patients. The DSAA is an obligatory national quality registry that started in 2013, which registers all patients who undergo aortic aneurysm repair in the Netherlands. Cases were women who died in hospital or within 90 days of primary elective OSR, and controls were women who survived the first 90 days post-operatively, treated in the same hospitals as the cases. Data for this study were subsequently collected from the individual patients’ medical files. Centres with no peri-operative deaths among women after OSR or centres that declined participation were excluded. It was reasoned that using controls from the same hospital as cases would account for similar exposure to unmeasured hospital related confounders, such as referral patterns, patient baseline characteristics, skills of the surgical team, and quality of post-operative care. Further exclusion criteria are shown in Fig. 1. The study and the preparation of the manuscript were done in accordance with the Strengthening the Reporting of Observational Studies in Epidemiology (STROBE) statement.^8^

### Variables and definitions

This exploratory study aimed to identify risk factors associated with peri-operative death. Therefore, a wide variety of pre-, intra-, and post-operative variables was collected from the patients’ medical files. Definitions of pre-operative variables are provided in Supplementary Table S1. Symptomatic peripheral arterial disease (PAD) was defined as Fontaine classification II – IV or prior treatment for PAD. Computed tomography angiograms done prior to OSR were reviewed by V.N.T., M.J.W.K., and local vascular surgeons to assess the patency of the coeliac artery (CA), and the superior (SMA) and inferior mesenteric arteries (IMA), blinded to the eventual outcome of death or survival and BI. Prior to data collection, the assessment of the mesenteric arteries was standardised with the help of S.M. Schreuder (interventional radiologist Amsterdam UMC), to limit variation in data collection across all centres. The degree of CA and SMA stenosis was expressed in percentages and calculated as follows: the diameter (measured perpendicularly from sagittal sections) of the mesenteric artery at the segment where any stenosis was present, divided by the diameter of the more distal mesenteric artery segment without stenosis. If sagittal sections gave insufficient information, axial and or coronal sections were used. A stenosis of >70% was considered as significant. The IMA was assessed as either patent or occluded on axial sections. The definitions of intra- and post-operative variables are shown in the corresponding tables. Any uncertainties in the data collection were resolved with M.J.W.K. and vascular surgeons from the participating centres.

### Endpoints

The endpoints were factors associated with 90 day or in hospital death after elective AAA OSR. Ninety day survival status was confirmed in the patient file during hospital follow up, or through consultation of the Personal Records Database via the participating centre.

### Statistical analysis

Categorical variables were reported as absolute numbers and proportions. Continuous variables were presented as mean ± standard deviation or as median (interquartile range), depending on the distribution. To assess differences between the cases and controls, the chi square or Fisher's exact test were used for categorical variables, as appropriate. The Student's *t* test or Mann–Whitney *U* test were used for continuous variables following a normal or skewed distribution, respectively. In a pre-defined analysis, cases were compared with age matched controls to account for age as important risk factor for death.^4^^,^^9^ Cases were compared with multiple controls to improve the power to detect differences between the groups as the number of cases in the study was small. A ratio of 1:3 was used as it retained the most cases with age matched controls. Cases were matched with controls of the same age at the time of AAA repair, without replacement of cases.

Univariable logistic regression analysis was conducted to express the association between variables with death as crude OR and 95% CI. Categorical variables with more than two groups (smoking, chronic obstructive pulmonary disease [COPD], PAD, and post-operative complications) were dichotomised (yes or no) as the number of events was insufficient to calculate the OR for each separate group. Variables with a significant association on univariable analysis (*p* < .10) and or those considered clinically relevant were entered into a multivariable logistic regression analysis to identify variables statistically significantly associated with death (adjusted OR with 95% CI). Conditional logistic regression analyses were performed for the cases and age matched control group. Two separate multivariable logistic regression analyses were conducted to assess risk factors associated with death in two phases of AAA care: the pre-operative phase comprising pre-operative risk factors, and the operative phase consisting of intra-operative factors and post-operative complications. The Nagelkerke pseudo *R*^*2*^ value generated in logistic regression analysis can attain a value of 0–1. A value closer to1 indicates better explanation of the mortality risk by the variables in the model. Statistical analyses were performed with SPSS Statistics version 26 (IBM, Armonk, NY, USA) or RStudio version 4.0.3 (The R Foundation for Statistical Computing, Vienna, Austria). To account for multiple testing, the *p* value was adjusted for statistical significance to <.01.

## Results

### Study population

In total, 309 women who underwent elective OSR for AAA in 21 centres (2013–2019) were identified through the DSAA registry (Fig. 1). Three centres declined participation in the current study and 32 hospitals were excluded because they had no deaths in women after elective OSR (no cases). Forty-three patients were excluded, the majority because they had a symptomatic or ruptured AAA. Eventually, 266 women were included, comprising 30 cases and 236 controls. The majority of patients had an infrarenal AAA (cases 73.3%, controls 68.1%; *p* = .68). Other patients had a juxtarenal (cases 26.7%, controls 30.6%; *p* = .68) or suprarenal AAA (cases 0%, 1.3% controls; *p* = 1.0). The main reason for OSR was non-suitability for EVAR (cases 92.9%, controls 88.1%).

### Pre-operative determinants

The demographics of cases and controls are provided in Table 1. Cases were statistically significantly older than controls at the time of surgery and more often had symptomatic PAD. The prevalence of COPD was higher in the cases.

**Table 1:** Patient characteristics and mesenteric artery patency on pre-operative computed tomography angiography of women who underwent elective open surgical repair of abdominal aortic aneurysm (AAA).

Characteristic	Cases *vs.* controls				Cases *vs.* age matched controls (ratio 1:3)			
	Total group (*n* = 266)	Cases (*n* = 30)	Controls (*n* = 236)	*p* value	Total group (*n* = 104)	Cases (*n* = 26)	Controls (*n* = 78)	*p* value
Age – y	72 (67.0, 77.0)	75 (71.0, 78.3)	71 (66.0, 77.0)	.002	75 (71.0, 77.0)	75 (71.0, 77.3)	75 (71.0, 77.0)	–
AAA diameter – mm	56 (52.0, 62.0)	55 (51.8, 60.3)	56 (52.0, 62.0)	.35	56 (52.0, 62.0)	55 (51.8, 60.3)	57 (52.0, 63.0)	.12
*AAA morphology*†				.78				.69
Infrarenal	182 (68.4)	22 (73)	160 (67.8)		68 (65.4)	18 (69)	50 (64)	
Juxtarenal	80 (30.1)	8 (27)	72 (30.5)		35 (33.6)	8 (31)	27 (35)	
Suprarenal	3 (1.1)	0 (0)	3 (1.3)		0 (0.0)	0 (0)	0 (0)	
Diabetes mellitus†	45 (16.9)	6 (20)	39 (16.5)	.80	16 (15.4)	6 (23)	10 (13)	.22
Hypertension†	183 (68.8)	24 (80)	159 (67.4)	.29	72 (69.2)	20 (77)	52 (67)	.33
*Smoking history*†				.43∗				.20∗
None or none within last 10 years	45 (16.9)	3 (10)	42 (17.8)		20 (19.2)	3 (11)	17 (22)	
None current but within last 10 years	70 (26.3)	7 (23)	63 (26.7)		26 (25.0)	6 (23)	20 (26)	
Current smoker	137 (51.5)	18 (60)	119 (50.4)		50 (48.1)	15 (58)	35 (45)	
Coronary artery disease†	75 (28.2)	12 (40)	63 (26.7)	.13	35 (33.7)	11 (42)	24 (31)	.27
Congestive heart failure	12 (4.5)	2 (7)	10 (4.2)	.63	5 (4.8)	2 (8)	3 (4)	.38
*Chronic obstructive pulmonary disease*†				.023∗				.088∗
Gold I	19 (7.1)	2 (6.7)	17 (7.2)		9 (8.7)	2 (7.7)	7 (9.0)	
Gold II	33 (12.4)	7 (23.3)	26 (11.0)		13 (12.5)	5 (19.2)	8 (10.3)	
Gold III	11 (4.1)	3 (10.0)	8 (3.4)		3 (2.9)	2 (7.7)	1 (1.3)	
Gold IV	2 (0.8)	0 (0.0)	2 (0.8)		2 (1.9)	0 (0.0)	2 (2.6)	
Gold unspecified	25 (9.4)	5 (16.7)	20 (8.5)		10 (9.6)	5 (19.2)	5 (6.4)	
*Renal disease*†				.58				.73
eGFR <15	1 (0.4)	0 (0.0)	1 (0.4)		1 (1.0)	0 (0.0)	1 (1.3)	
eGFR 15-<30	6 (2.3)	1 (3.3)	5 (2.1)		4 (3.8)	1 (3.8)	3 (3.8)	
eGFR 30-60	78 (29.3)	10 (33.3)	68 (28.8)		30 (28.8)	7 (26.0)	23 (29.5)	
eGFR >60	172 (64.7)	18 (60.0)	154 (65.3)		64 (61.5)	17 (65.4)	47 (60.3)	
*Peripheral arterial disease*†				.002∗				.009∗
Fontaine stage I	12 (4.5)	0 (0)	12 (5.1)		8 (7.7)	0 (0)	8 (10)	
Fontaine stage II	51 (19.2)	11 (37)	40 (16.9)		23 (22.1)	10 (38)	13 (17)	
Fontaine stage III	3 (1.1)	0 (0)	3 (1.3)		0 (0.0)	0 (0)	0 (0)	
Fontaine stage IV	3 (1.1)	0 (0)	3 (1.3)		1 (1.0)	0 (0)	1 (1)	
Fontaine stage unspecified	6 (2.3)	3 (10)	3 (1.3)		4 (3.8)	3 (11)	1 (1)	
Transient ischaemic attack or stroke†	38 (14.3)	4 (13)	34 (14.4)	1.0	12 (11.5)	4 (15)	8 (10)	.50
Previous abdominal surgery†	105 (39.5)	13 (43)	92 (39.0)	.69	40 (38.5)	12 (46)	28 (36)	.30
*Mesenteric artery patency*								
Coeliac artery				.016				.10
*Stenosis <70%*	234 (88.0)	22 (73)	212 (89.8)		84 (80.8)	18 (69)	66 (85)	
*Stenosis >70%*	32 (12.0)	8 (27)	24 (10.2)		20 (19.2)	8 (31)	12 (15)	
Superior mesenteric artery				.025				.06
*Stenosis <70%*	255 (95.9)	26 (87)	229 (97.0)		97 (93.3)	22 (85)	75 (96)	
*Stenosis >70%*	11 (4.1)	4 (13)	7 (3.0)		7 (6.7)	4 (15)	3 (4)	
Inferior mesenteric artery†				1.0				.81
*Patent*	135 (50.8)	16 (53)	119 (50.4)		45 (43.3)	12 (46)	33 (42)	
*Occluded*	126 (47.4)	14 (47)	112 (47.5)		57 (54.8)	14 (54)	43 (55)	

Categorical data are presented as n (%) and continuous variables as median (interquartile range).

∗ *p* value represents a comparison of dichotomous groups: smoking history (yes/no), COPD (yes/no), symptomatic peripheral arterial disease (Fontaine II, III, IV, and unspecified classification but treated) *vs*. asymptomatic (Fontaine I or no peripheral arterial disease) and medication use (yes/no). Medications that were used are shown in Supplementary Table S2.

† Missing data can be found in Supplementary Table S3.

### Mesenteric artery stenosis

Cases more often had >70% stenosis of the CA and SMA than controls (Table 1). The association with BI of a CA stenosis >70% (OR 2.20, 95% CI 0.82–5.92; *p* = .12) or SMA stenosis >70% (OR 5.23, 95% CI 1.43–19.13; *p* = .012) was not statistically significant. Furthermore, > 70% stenosis of either the CA or SMA with a patent IMA (*vs.* occluded IMA) was not associated with BI (OR 1.09 [95% CI 0.06–19.63; *p* = .95] and infinite OR [95% CI 0.00 – infinity; *p* = 1.0], respectively).

Of all cases, three (10%) had a significant stenosis of both CA and SMA compared with two (0.8%) controls (*p* = .011). These three patients died as a result of BI, and the two controls had no complications. A stenosis of >70% of both the CA and SMA was associated with BI (OR 13.5, 95% CI 2.16–84.54; *p* = .005) and death (OR 13.00, 95% CI 2.08–81.28; *p* = .006).

### Operative determinants

The intra-operative characteristics and post-operative complications are listed in Table 2, Table 3, respectively. Cases had more intra-operative blood loss than controls. Suprarenal clamping was not more often necessary in cases than in controls, yet the clamp time was longer. Cases more often had the complications of acute MI (AMI), renal failure, and BI than controls.

**Table 2:** Intra-operative characteristics of women who underwent elective open surgical repair of abdominal aortic aneurysm.

	Cases *vs.* controls				Cases *vs.* age matched controls (ratio 1:3)			
	Total group (*n* = 266)	Cases (*n* = 30)	Controls (*n* = 236)	*p* value	Total group (*n* = 104)	Cases (*n* = 26)	Controls (*n* = 78)	*p* value
*Type of graft*				.439				.523
Tube	149 (56.0)	19 (63)	130 (55.1)		58 (55.8)	16 (61)	42 (54)	
Bifurcated	117 (44.0)	11 (37)	106 (44.9)		46 (44.2)	10 (38)	36 (46)	
Estimated blood loss – dL∗^,^†	130 (70–180)	156 (108–300)	120 (68–180)	.007	130 (70–240)	156 (98–300)	110 (60–175)	.041
Operation duration – min†	190 (150.0–233.0)	217 (161.3–256.0)	187 (149.0–226.5)	.130	194 (149.0–243.0)	217 (161.3–256.0)	192 (143.8–236.5)	.324
*Clamping site*†				.499‡				.573
Suprarenal above 1/2 arteries	1 (0.4)	1 (3)	0 (0.0)		1 (1.0)	1 (4)	0 (0)	
Suprarenal above 1 artery	41 (15.4)	4 (13)	37 (15.7)		18 (17.3)	4 (15)	14 (18)	
Suprarenal above 2 arteries	45 (16.9)	6 (20)	39 (16.5)		20 (19.2)	6 (23)	14 (18)	
Infrarenal	168 (63.2)	17 (57)	151 (64.0)		57 (54.8)	13 (50)	44 (56)	
Proximal clamping time – min†^,^§	29 (21.0–36.8)	35 (30.3–58.8)	26 (20.3–35.0)	.016	30 (22.0–38.0)	35 (30.3–58.8)	30 (21.0–36.0)	.041
Heparin administration†	239 (89.8)	27 (90)	212 (89.8)	.576	92 (88.5)	24 (92)	68 (87)	.830

Categorical data are presented as *n* (%) and continuous variables are shown as median (interquartile range).

∗ Estimated blood loss without use of packed cells, plasma, or cell saver.

† Missing data can be found in Supplementary Table S3.

‡ Suprarenal clamping *vs*. infrarenal clamping.

§ Only applicable for suprarenal clamping.

**Table 3:** Post-operative complications of women who underwent elective open surgical repair of abdominal aortic aneurysm (AAA).

	Cases *vs.* controls				Cases *vs.* age matched controls (ratio 1:3)			
	Total group (*n* = 266)	Cases (*n* = 30)	Controls (*n* = 236)	*p* value∗	Total group (*n* = 104)	Cases (*n* = 26)	Controls (*n* = 78)	*p* value∗
*Acute myocardial infarction*				<.001				.003
Condition did not occur	248 (93.2)	20 (67)	228 (96.6)		92 (88.5)	18 (69)	74 (95)	
Little or no haemodynamic consequence	3 (1.1)	1 (3)	2 (0.8)		0 (0)	0 (0)	0 (0)	
Acute myocardial infarction requiring percutaneous coronary intervention	6 (2.3)	1 (3)	5 (2.1)		4 (3.8)	1 (4)	3 (4)	
Severe haemodynamic consequences requiring resuscitation, cardiac arrest, or fatal outcome	9 (3.4)	8 (27)	1 (0.4)		8 (7.7)	7 (27)	1 (1)	
*Renal failure*				<.001				.001
Condition did not occur	216 (81.2)	13 (43)	203 (86.0)		75 (72.1)	11 (42)	64 (82)	
No dialysis	35 (13.2)	11 (37)	24 (10.2)		23 (22.1)	10 (38)	13 (17)	
Renal failure requiring temporary dialysis with permanently reduced renal function requiring surveillance by a nephrologist	8 (3.0)	1 (3)	7 (3.0)		1 (1.0)	1 (4)	0 (0)	
Renal failure requiring permanent dialysis, transplant or fatal outcome	7 (2.6)	5 (17)	2 (0.8)		5 (4.8)	4 (15)	1 (1)	
*Bowel ischaemia*†				<.001				<.001
Condition did not occur	236 (88.7)	12 (40)	224 (94.9)		85 (81.7)	10 (38)	75 (96)	
Recovered without intervention	4 (1.5)	0 (0)	4 (1.7)		0 (0.0)	0 (0)	0 (0)	
Recovered with intravenous antibiotics or total parenteral nutrition	1 (0.4)	0 (0)	1 (0.4)		1 (1.0)	0 (0)	1 (1)	
Bowel resection or fatal outcome	24 (9.0)	17 (57)	7 (3.0)		17 (16.3)	15 (58)	2 (3)	

Data are presented as *n* (%). Groups of complications that are considered major or minor according to vascular surgeons, on which they reached consensus for AAA treatment, were analysed.^33^ Only complications that were statistically significant are shown. Five of the cases that developed acute myocardial infarction had a fatal outcome (four in age matched analysis), and 13 of those who developed bowel ischaemia had a fatal outcome (11 in the age matched analysis).

∗ *p* value represents a comparison between the dichotomous groups yes or no.

† Missing data can be found in Supplementary Table S3. Complications that were not statistically significantly different between groups included congestive heart failure, pulmonary embolism, cerebrovascular complication, post-operative haemorrhage, thromboembolic event, spinal cord ischaemia, and wound infection.

Intra-operative blood loss was not associated with AMI (OR 1.001, 95% CI 0.997–1.005; *p* = .55), but was associated with BI (OR 1.006, 95% CI 1.003–1.009; *p* < .001).

### Risk factors associated with death

The results of the multivariable analyses are shown in Table 4 and indicate the significant association between age and symptomatic PAD and mortality (Nagelkerke *R*^*2*^ = 0.194). Multivariable analysis of operative risk factors demonstrated the significant associations of AMI and BI with death (Nagelkerke *R*^*2*^ = 0.559).

**Table 4:** Logistic regression analysis of risk factors for 90 day mortality in women undergoing elective open surgical repair of abdominal aortic aneurysm.

	Cases *vs.* controls				Cases *vs.* age matched controls (ratio 1:3)			
	Univariable analysis OR (95% CI)	*p* value	Multivariable analysis OR (95% CI)	*p* value	Univariable analysis OR (95% CI)	*p* value	Multivariable analysis OR (95% CI)	*p* value
*Pre-operative variables*								
Age	1.10 (1.03–1.18)	.003	1.11 (1.03–1.19)	.005∗	–	–	–	–
Chronic obstructive pulmonary disease	2.60 (1.16–5.86)	.021	2.42 (0.99–5.89)	.051∗	2.16 (0.89–5.23)	.088	2.71 (0.92–7.99)	.071∗
Peripheral arterial disease†	3.39 (1.53–7.50)	.003	3.91 (1.57–9.74)	.003∗	3.57 (1.37–9.30)	.009	4.55 (1.21–17.15)	.025∗
Superior mesenteric artery stenosis >70% *vs.* <70%	5.03 (1.38–18.35)	.014	2.55 (0.46–14.20)	.285∗	5.16 (0.92–28.83)	.061	1.69 (0.00–infinity)	.999∗
*Intra-operative variables*								
Estimated blood loss – dL	1.005 (1.002–1.008)	<.001	1.003 (0.999–1.006)	0.188‡	1.0033 (1.0001–1.0065)	.041	1.000 (0.996–1.005)	0.909‡
*Post-operative complications*								
Acute myocardial infarction	14.25 (5.06–40.16)	<.001	38.94 (9.63–157.45)	<.001‡	7.41 (1.95–28.17)	.003	9.34 (1.66–52.42)	.011‡
Renal failure	8.04 (3.58–18.09)	<.001	2.36 (0.75–7.40)	.140‡	5.39 (2.04–14.27)	.001	1.58 (0.38–6.49)	.529‡
Bowel ischaemia	26.44 (10.33–67.69)	<.001	27.99 (7.77–100.88)	<.001‡	41.10 (5.41–312.26)	<.001	35.59 (3.41–370.95)	.003‡

Variables used in multivariable logistic regression analysis are shown. Categorical variables with more than 2 groups were categorised into the dichotomous groups yes or no.

∗ *p* value corrected for age (except in age matched analysis), chronic obstructive pulmonary disease, peripheral arterial disease and superior mesenteric artery stenosis.

† Peripheral arterial disease: symptomatic (Fontaine II, III, IV, and unspecified classification but treated) *vs*. asymptomatic. Thirty patients in the case control analysis and 26 in the age matched analysis died.

‡ *p* value corrected for intra-operative blood loss, acute myocardial infarction, renal failure, and bowel ischaemia.

### Age matched case control analysis

The age matched sub-analysis included 26 cases and 78 controls. The median (IQR) patient age was 75 (71, 77) years and the prevalence of symptomatic PAD was statistically significantly higher in cases than controls (Table 1). Intra-operative blood loss was higher in cases than in controls but the difference was not statistically significant (Table 2). The complications AMI, renal failure and BI were significantly more prevalent in cases than in controls (Table 3).

While in univariable analysis symptomatic PAD was associated with death, it lost its statistical significance in multivariable analysis. In the multivariable analysis of operative risk factors, AMI and BI were statistically significantly associated with death (Table 4).

## Discussion

In this multicentre case control study in women undergoing elective AAA OSR, it was found that older age and symptomatic PAD were associated with increased 90 day mortality risk and that AMI and BI increased the risk of peri-operative death. Intra-operative blood loss was associated with BI and, although not statistically significant, severe SMA stenosis had a clinically relevant association with BI.

Advanced age is a well known risk factor for peri-operative death after AAA surgery,^10^, ^11^, ^12^, ^13^ and particularly dangerous when accompanied by comorbidity. The impact of comorbidities was demonstrated in a study showing a 14.8% mortality risk after AAA repair in octogenarians with comorbidities *vs.* 3.8% in octogenarians without comorbidities.^10^

In this study, PAD emerged as risk factor for death, corroborating previous findings.^14^, ^15^, ^16^ Symptomatic PAD is an indication of severe systemic atherosclerotic disease and is associated with coronary artery disease (CAD) and cerebrovascular disease.^17^^,^^18^ However, no association between symptomatic PAD and AMI related death (OR 1.77, 95% CI 0.25–12.60; *p* = .57) could be demonstrated in this sample.

In this study, CAD was not more prevalent in cases than controls, and it was also not associated with death. As the detection of CAD is challenging in women,^19^ it may be that coronary comorbidity could not be adequately accounted for. Currently, there is insufficient evidence to suggest that cardiopulmonary exercise testing or cardiac stress testing in patients prior to undergoing AAA repair improves risk stratification, although specific data for women are lacking.^20^, ^21^, ^22^ Also, there is no compelling evidence that pre-operative coronary revascularisation reduces peri-operative cardiac death and morbidity.^23^ The question remains how the incidence of post-operative AMI can be lowered. In the last two decades no studies investigating the benefit of pre-operative coronary revascularisation have been published. Together with the improved understanding of sex related differences in cardiac disease, cardiac evaluation in women prior to elective OSR might be improved and could be meaningful in reducing the mortality risk.

As 20% of deaths after AAA OSR can be attributed to BI, every effort to prevent this complication is crucial.^24^ The finding that intra-operative blood loss is associated with post-operative BI is not novel,^25^ yet it may partly explain the higher risk of BI in cases. The Dutch Society for Vascular Surgery recommends that a team of two vascular surgeons performs the operation. Although not supported by scientific evidence, this may help prevent the sequelae that probably start with excessive blood loss. It is well known that women have a high risk of BI after open AAA repair,^25^^,^^26^ which is why a full assessment of the visceral arteries was done. No significantly increased risks in patients with >70% CA or >70% SMA stenosis with or without a patent IMA were found. However, an association between SMA stenosis >70% and BI was found, but it was not statistically significant (OR 5.23, 95% CI 1.43–19.13; *p* = .012) because of the adjusted *p* value; it is also possible that the sample was too small to achieve statistical significance. Although a rare condition, concomitant CA and SMA stenosis >70% increased the risk of BI and death significantly. The European Society for Vascular Surgery guidelines for AAA management do not give recommendations for prophylactic SMA revascularisation in patients undergoing OSR,^27^ and this was not done pre- or intra-operatively in any of the study patients. Revascularisation prior to or during open AAA repair should be investigated in future studies to determine whether this is helpful in preventing BI. Unfortunately, data on IMA replantation were not recorded. Although there is debate as to whether IMA replantation prevents BI, it might be beneficial in selected cases, such as older patients.^28^ No association between IMA patency in patients with a single CA or SMA stenosis and BI was found, suggesting that the IMA is not a relevant risk factor in BI. Moreover, the status of the hypogastric arteries as a factor contributing to BI was not assessed, limiting the understanding of why BI occurred in some women.

The 7.3% peri-operative mortality risk is not unique to the Netherlands and has been reported to vary in other countries (between 6.9% and 9.5%).^3^^,^^4^^,^^9^ The aim was to identify risk factors specifically for women that could be targeted to optimise pre-operative management and lower the mortality rate. It is disappointing that, in the end, only well known risk factors were found, possibly because only what is considered to be relevant was measured. Therefore, it might be better to look beyond the unknown and in other fields, for example in pharmacology, where it has been found that differences in pharmacokinetics and pharmacodynamics contribute to the higher susceptibility of women than men to adverse drug reactions.^29^^,^^30^ Another factor suggested to affect mortality risk is service provision. Indeed, an inverse relationship between OSR hospital volume and death is also seen in the Netherlands.^31^ However, in this study, after accounting for hospital volume, female sex remained independently associated with death, suggesting that an explanation is more likely to be found at patient level.^31^

There are several limitations to this study. Owing to the retrospective design, the authors were limited to the available information. However, the proportion of missing data was low.

As mentioned previously, data on IMA re-implantation or the status of the hypogastric arteries were not recorded, which would have enabled a better understanding of the occurrence of BI. Another limitation is that frailty status was not recorded; frailty has been found to be associated with death after OSR.^32^

As this was an exploratory study and although a lower *p* value was used, the results should be interpreted with caution. The study sample size was small and dependent on the number of patients treated with elective OSR between 2013 and 2019 in the Netherlands. Consequently, this study may have lacked sufficient statistical power to find relevant differences between cases and controls. The number of variables that could be entered in the multivariable analysis was also limited due to the number of cases.

Because of the relatively low prevalence of AAA in women, international collaboration in a large prospective study is necessary to increase the sample size and produce more robust evidence to predict the mortality risk in women after AAA OSR.

Finally, by selecting controls from the same hospital as cases, the aim was to account for biases as much as possible. The disadvantage of excluding centres with zero deaths is that an opportunity to compare pre- and intra-operative characteristics of the women treated in such centres was missed, and therefore the study population may not represent the complete Dutch female population undergoing elective AAA OSR.

### Conclusion

By evaluating a wide variety of variables, the aim was to identify factors in women associated with a high peri-operative mortality risk. It is disappointing that this study only confirmed that older age and symptomatic PAD are risk factors for peri-operative death in women undergoing AAA OSR, and that there is an increased risk of death associated with AMI and BI.

One of the measures used to prevent BI is to limit intra-operative blood loss as much as possible. The clinically relevant finding that a >70% SMA stenosis was associated with an increased risk of BI must be considered a call for action in selected cases and for future studies. Although rare, concomitant CA and SMA stenosis >70% seems to be a dangerous combination and may need treatment before OSR, but this must be proven first in future studies.

## Conflicts of interest

None.

## Funding

The study was funded by the AMC Foundation. The funder was not involved in the study design, data collection, data analysis, interpretation of the results, or writing of the manuscript.

## Appendix A

∗**Study Group Collaborators in alphabetical order:**

G.P. Akkersdijk^c^, G.J. Boer^c^, L.H. Bouwman^d^, J. Diks^e^, J.W. Elshof^f^, R.H. Geelkerken^g,h^, G.H. Ho^i^, P.T. den Hoed^j^, B.P. Keller^k^, J.W. Klunder^l^, O.H. Koning^m^, R.R. Kruse^n^, J.H. Lardenoye^o^, M.S. Lemson^p^, S.J.G. Leeuwerke^g^, F.T. van der Linden^q^, M.E. Pierie^r^, H.P. van ’t Sant^s^, O. Schouten^t^, S.M. Schreuder^u^, R.M. The^l^, L. van Silfhout^p^, R.B. van Tongeren^v^, P.W. Vriens^w^, A.M. Wiersema^x^, A. Wiersma^k^, C.J. Zeebregts^y^

^c^Department of Surgery, Maasstad Hospital, Rotterdam, the Netherlands.

^d^Department of Surgery, Zuyderland, Sittard-Geleen, Heerlen and Brunssum, the Netherlands.

^e^Department of Surgery, Bravis Hospital, Bergen op Zoom and Roosendaal, the Netherlands.

^f^Department of Surgery, VieCuri Hospital, Venlo and Venray, the Netherlands.

^g^Department of Vascular Surgery, Medisch Spectrum Twente, Enschede, the Netherlands

^h^Multi-Modality Medical Imaging Group, Techmed Center, University of Twente, Enschede, the Netherlands

^i^Department of Surgery, Amphia Hospital, Breda, the Netherlands.

^j^Department of Surgery, Ikazia Hospital, Rotterdam, the Netherlands.

^k^Department of Surgery, Martini Hospital, Groningen, the Netherlands.

^l^Department of Surgery, St. Jansdal Hospital, Harderwijk, the Netherlands.

^m^Department of Surgery, Jeroen Bosch Ziekenhuis, ’s Hertogenbosch, the Netherlands.

^n^Department of Surgery, Ziekenhuis Groep Twente, Hengelo and Almelo, the Netherlands.

^o^Department of Surgery, Rijnstate Hospital, Arnhem, the Netherlands.

^p^Department of Surgery, Slingeland Hospital, Doetinchem, the Netherlands.

^q^Department of Surgery, St. Anna Hospital, Geldrop and Eindhoven, the Netherlands.

^r^Department of Surgery, Isala Hospital, Zwolle, the Netherlands.

^s^Department of Surgery, OLVG, Amsterdam, the Netherlands.

^t^Department of Surgery, Reinier de Graaf Gasthuis, Delft, the Netherlands.

^u^Department of Radiology, Amsterdam University Medical Center, University of Amsterdam, Amsterdam, the Netherlands.

^v^Department of Surgery, Deventer Hospital, Deventer, the Netherlands.

^w^Department of Surgery, Elizabeth TweeSteden Hospital, Tilburg, the Netherlands.

^x^Department of Surgery, Dijklander Hospital, Purmerend, the Netherlands.

^y^Department of Surgery (Division of Vascular surgery), University Medical Center of Groningen, University of Groningen, Groningen, the Netherlands.
