# Supplementary material for: Identifying Women at High Risk of 90 Day Death after Elective Open Abdominal Aortic Aneurysm Repair: A Multicentre Case Control Study
Source: EJVES Vasc Forum. 2022 Nov 9;57:17–27. doi: 10.1016/j.ejvsvf.2022.10.005 (PMC9712556; doi:10.1016/j.ejvsvf.2022.10.005)
Supplement: Multimedia component 1 [file mmc1.pdf]

Supplementary Table S1. Preoperative variables and their corresponding definitions

Preoperative variables	Definitions
**Age**	Age at the time of AAA surgery in years.
**AAA diameter**	Maximum anterior-posterior diameter of the aortic aneurysm at the time of surgery in mm on CTA.
**AAA morphology**	Infrarenal = Abdominal aortic aneurysm below the renal arteries<br>Juxtarenal = Abdominal aortic aneurysm below and without the involvement of the renal arteries, and suprarenal aortic cross-clamping is required for surgical repair<br>Suprarenal = Abdominal aortic aneurysm with the involvement of the renal arteries with/without involvement of the mesenteric arteries
**Diabetes mellitus**	Any history of diabetes or current diabetes, treated (with hypoglycaemic agents) or not.
**Hypertension**	Hypertension previously or currently treated with antihypertensive drugs.
**Smoking history**	Categorised in (1) none or none for last 10 years, (2) none current, but smoked in last 10 years, (3) current smoker.
**Coronary artery disease**	Including any of the following:<br>1 = Angina pectoris, stable angina: angina necessitating episodic or permanent medication use;<br>2 = History of unstable angina;<br>3 = Previous myocardial infarction;<br>4 = History of percutaneous coronary intervention (PCI);<br>5 = History of coronary artery bypass graft surgery (CABG)
**Congestive heart failure**	The presence of signs and symptoms of either right or left ventricular failure or both and the diagnosis should be confirmed by non-invasive or hemodynamic measurements.
**COPD**	1 = Gold I: mild, more of equivalent to 80% predicted FEV1 value<br>2 = Gold II: moderate, between 50-80% predicted FEV1 value<br>3 = Gold III: severe, between 30-50% predicted FEV1 value<br>4 = Gold IV: very severe, less than 30% predicted FEV1 value<br>5 = Gold non-specified
**Renal disease**	The absolute value of the registered eGFR assessed preoperatively will be collected.
**Peripheral arterial disease**	Fontaine classification<br>1 = Stage I: Asymptomatic<br>2 = Stage II: Intermittent claudication<br>3 = Stage III: Ischaemic rest pain<br>4 = Stage IV: Ulceration or gangrene, or both<br>If the Fontaine Classification was unknown, this was considered clinically relevant if one underwent any of the following interventions: supervised exercise training, percutaneous transluminal angioplasty, or peripheral vascular surgery.
**TIA or ischemic stroke**	Diagnosis of TIA or stroke mentioned in a hospital report.
**Previous abdominal surgery**	Any surgery in the abdomen previous to the open surgical repair for the abdominal aortic aneurysm.
