# Supplementary material for: Identifying Women at High Risk of 90 Day Death after Elective Open Abdominal Aortic Aneurysm Repair: A Multicentre Case Control Study
Source: EJVES Vasc Forum. 2022 Nov 9;57:17–27. doi: 10.1016/j.ejvsvf.2022.10.005 (PMC9712556; doi:10.1016/j.ejvsvf.2022.10.005)
Supplement: Multimedia component 2 [file mmc2.pdf]

Supplementary Table S2. Medication of women who underwent elective open surgical repair of abdominal aortic aneurysm

	Cases versus controls				Cases versus age-matched controls (ratio of 1:3			
	Total group (n=266)	Cases (n=30)	Controls (n=236)	*P* value	Total group (n=104)	Cases (n=26)	Controls (n=78)	*P* value
**Antihypertensive agents**				0.657*				0.773*
B blockers	121 (45.5)	13 (43.3)	108 (45.8)		46 (44.2)	11 (42.3)	35 (44.9)	
ACE inhibitors or ARB with/without diuretic	117 (44.0)	14 (46.7)	103 (43.6)		44 (42.3)	13 (50.0)	31 (39.7)	
Diuretics	57 (21.4)	10 (33.3)	47 (19.9)		21 (20.2)	7 (26.9)	14 (17.9)	
Calcium channel blockers with/without ARB	74 (27.8)	7 (23.3)	67 (28.4)		26 (25.0)	7 (26.9)	19 (24.4)	
**Glucose-lowering agents**				1.000*				0.706*
Metformin	21 (7.9)	3 (10.0)	18 (7.6)		7 (6.7)	3 (11.5)	4 (5.1)	
Insulin	5 (1.9)	0 (0.0)	5 (2.1)		1 (1.0)	0 (0.0)	1 (1.3)	
Sitagliptin	1 (0.4)	0 (0.0)	1 (0.4)		0 (0.0)	0 (0.0)	0 (0.0)	
**Anticoagulant agents**				1.000*				0.648*
Antiplatelet drugs	197 (74.1)	21 (70.0)	176 (74.6)		77 (74.0)	17 (65.4)	60 (76.9)	
Vitamin K antagonists	26 (9.8)	4 (13.3)	22 (9.3)		10 (9.6)	4 (15.4)	6 (7.7)	
NOAC	3 (1.1)	1 (3.3)	2 (0.8)		1 (1.0)	1 (3.8)	0 (0.0)	
**Statins**				0.657*				0.714*
Simvastatin	107 (40.2)	15 (50.0)	92 (39.0)		41 (39.4)	12 (46.2)	29 (37.2)	
Pravastatin	11 (4.1)	0 (0.0)	11 (4.7)		5 (4.8)	0 (0.0)	5 (6.4)	
Rosuvastatin	22 (8.3)	2 (6.7)	20 (8.5)		8 (7.7)	2 (7.7)	6 (7.7)	
Atorvastatin	55 (20.7)	7 (23.3)	48 (20.3)		21 (20.2)	6 (23.1)	15 (19.2)	
**Cardiac medication**				0.314*				0.417*
Digoxin	6 (2.3)	2 (6.7)	4 (1.7)		3 (2.9)	2 (7.7)	77 (98.7)	
Amiodarone	2 (0.8)	0 (0.0)	2 (0.8)		2 (1.9)	0 (0.0)	1 (1.3)	
**Immunosuppressive agents**				0.205*				0.245*
Prednisone	17 (6.4)	2 (6.7)	15 (6.4)		8 (7.7)	2 (7.7)	6 (7.7)	
Beclomethasone	5 (1.9)	1 (3.3)	4 (1.7)		1 (1.0)	1 (3.8)	0 (.0)	
Budesonide	1 (0.4)	1 (3.3)	0 (0.0)		1 (1.0)	1 (3.8)	0 (0.0)	
Azathioprine	1 (0.4)	0 (0.0)	1 (0.4)		1 (1.0)	0 (0.0)	1 (1.3)	
Methotrexate	2 (0.8)	0 (0.0)	2 (0.8)		0 (0.0)	0 (0.0)	0 (0.0)	
Other corticosteroids	3 (1.1)	0 (0.0)	3 (1.3)		2 (1.9)	0 (0.0)	2 (2.6)	
