# Supplementary material for: Identifying Women at High Risk of 90 Day Death after Elective Open Abdominal Aortic Aneurysm Repair: A Multicentre Case Control Study
Source: EJVES Vasc Forum. 2022 Nov 9;57:17–27. doi: 10.1016/j.ejvsvf.2022.10.005 (PMC9712556; doi:10.1016/j.ejvsvf.2022.10.005)
Supplement: Multimedia component 3 [file mmc3.pdf]

Supplementary Table S3. Missing data

Missing data of preoperative, intra-operative and post-operative complications are shown down below in *n* (%).

	**Cases versus controls**			**Cases versus age-matched controls (ratio of 1:3)**		
	Total group (n=266)	Cases (n=30)	Controls (n=236)	Total group (n=104)	Cases (n=26)	Controls (n=78)
**AAA morphology**	1 (0.4)	0 (0.0)	1 (0.4)	1 (1.0)	0 (0.0)	1 (1.3)
**Diabetes mellitus**	1 (0.4)	0 (0.0)	1 (0.4)	0 (0.0)	0 (0.0)	0 (0.0)
**Hypertension**	8 (3.0)	0 (0.0)	8 (3.4)	1 (1.0)	0 (0.0)	1 (1.3)
**Smoking history**	14 (5.3)	2 (6.7)	12 (5.1)	8 (7.7)	2 (7.7)	6 (7.7)
**Coronary artery disease**	10 (3.8)	1 (3.3)	9 (3.8)	3 (2.9)	1 (3.8)	2 (2.6)
**COPD**	42 (15.8)	2 (6.7)	40 (16.9)	19 (18.3)	2 (7.7)	17 (21.8)
**Renal disease**	9 (3.4)	1 (3.3)	8 (3.4)	5 (4.8)	1 (3.8)	4 (5.1)
**Peripheral arterial disease**	10 (3.8)	1 (3.3)	9 (3.8)	5 (4.8)	1 (3.8)	4 (5.1)
**Transient ischaemic attack or stroke**	1 (0.4)	0 (0.0)	1 (0.4)	1 (1.0)	0 (0.0)	1 (1.3)
**Previous abdominal surgery**	4 (1.5)	1 (3.3)	3 (1.3)	2 (1.9)	1 (3.8)	1 (1.3)
**Inferior mesenteric artery**	5 (1.9)	0 (0.0)	5 (2.1)	2 (1.9)	0 (0.0)	2 (2.6)
**Estimated blood loss**	19 (7.1)	0 (0.0)	19 (8.1)	7 (6.7)	0 (0.0)	7 (9.0)
**Operation duration**	39 (14.7)	4 (13.3)	35 (14.8)	14 (13.5)	4 (15.4)	10 (12.8)
**Clamping site**	11 (4.1)	2 (6.7)	9 (3.8)	8 (7.7)	2 (7.7)	6 (7.7)
**Proximal clamping time**	19 (21.8)	3 (27.3)	16 (21.1)	8 (20.5)	3 (27.3)	5 (17.9)
**Heparin administration**	20 (7.5)	2 (6.7)	18 (7.6)	9 (8.7)	1 (3.8)	8 (10.3)
**Bowel ischaemia**	1 (0.4)	1 (3.3)	0 (0.0)	1 (1.0)	1 (3.8)	0 (0.0)

Missing data statement. First, missing data for the whole study group were calculated, followed by the missing data of variables that were to be entered into multivariable analysis. For the multivariable analysis of preoperative risk factors, there were 3 missing values (2.5%) distributed over 3 cases (10.0%) and 49 missing values (5.2%) distributed over 46 controls (19.5%). For the multivariable analysis of operative risk factors, 1 case (3.3%) had 1 missing value (0.8%), and 19 controls (8.1%) had 19 missing values (2.0%). The missing data were considered minimal and at random. Therefore, a complete-case analysis was conducted.
